# Bulb-Priming Followed by Foliar Magnetite Nanoparticle Applications Improve Growth, Bulb Yield, Antioxidant Activities, and Iron Fortification in Shallot in Semi-Arid Regions

**DOI:** 10.3390/plants15020279

**Published:** 2026-01-16

**Authors:** Soroush Moguee, Sina Fallah, Lok R. Pokhrel, Zohrab Adavi

**Affiliations:** 1 Department of Agronomy, Faculty of Agriculture, Shahrekord University, Shahrekord P.O. Box 115, Iran; 2 Department of Public Health, The Brody School of Medicine, East Carolina University, Greenville, NC 27834, USA; 3 Department of Agriculture, Payame Noor University, Tehran P.O. Box 19395-3697, Iran

**Keywords:** bulb, cell ultrastructure, nanofertilizer, photosynthesis, secondary metabolite

## Abstract

Shallot (*Allium hirtifolium* Boiss.) is of considerable nutritional and medical significance due to its strong antioxidant properties; however, no nanophytotoxicity studies have assessed whether the use of nanofertilizers would improve shallot performance, micronutrient iron (Fe) enrichment, and yield in semi-arid regions. Herein, we evaluated the effects of magnetite nanoparticles (nFe_3_O_4_) on shallot grown for a full lifecycle in two semi-arid regions through bulb-priming followed by foliar application and compared them with conventional ferrous sulfate (FeSO_4_) fertilizer and untreated control. Our results showed remarkable cellular adaptations to semi-arid climate upon nFe_3_O_4_ treatment as leaves displayed thickened cell walls, distinct chloroplasts featuring organized thylakoid grana and stroma, normal mitochondria, abundant starch grains, and plastoglobuli around chloroplasts compared to FeSO_4_ or untreated control. At 900 mg/L nFe_3_O_4_, chlorophyll-a, chlorophyll-b, and carotenoid increased by 27–55%, 108–126%, and 77–97%, respectively, compared to FeSO_4_ applied at recommended field rate (1800 mg/L). Significant increments in bulb diameter (38–39%) and sister bulb number (300–500%) were observed upon 900 mg/L nFe_3_O_4_ treatment compared to FeSO_4_ (1800 mg/L) and control. Furthermore, with 900 mg/L nFe_3_O_4_ treatment, total phenol, flavonoids, and Fe in bulbs increased by 27–46%, 29–73%, and 486–549%, respectively, compared to FeSO_4_ (1800 mg/L). These findings demonstrate that bulb-priming followed by foliar application of 900 mg/L of nFe_3_O_4_ could significantly promote cellular adaptation, thereby improving photosynthetic efficiency, bulb yield, antioxidant activities, and Fe biofortification in shallot, and may serve as a novel approach for improving shallot production in semi-arid regions.

## 1. Introduction

Global food production has seen significant increase since the middle of the 20th century [1]. In the 21st century, rising global population, unequal economic progress, and worsening climate change have heightened attempts to improve crop production [2]. Nonetheless, as studies have progressed, the issue of “hidden hunger,” marked by a lack of vital micronutrients like iron (Fe) in the food supply, has received growing attention lately [3]. The health issues related to hidden hunger are a global challenge that hinders the realization of the United Nations Sustainable Development Goal 2 of achieving zero hunger by 2030 [4]. Agronomic biofortification is considered a crucial approach to tackle this health threat [5]. Considering the dynamics and bioavailability of micronutrients in soil, the application of micro/nanonutrient-based fertilizers via seed priming and foliar application has emerged as a cost-effective and environmentally sustainable method for achieving higher biofortification of essential micronutrients, such as Fe, in edible plant parts [6].

Iron deficiency remains a critical global health issue that affects millions of individuals across various demographics. According to recent estimates, approximately 1.62 billion people are afflicted by anemia, with Fe deficiency being the predominant cause in over 50% of these cases [7]. The World Health Organization (WHO) has reported that iron deficiency impacts nearly 30% of the world’s population, indicating a significant prevalence that poses a major public health challenge, particularly among vulnerable populations such as pregnant women and young children [8]. The widespread nature of this nutrient deficiency results in severe health consequences, including impaired cognitive and physical development in children, reduced work capacity in adults, and heightened susceptibility to infections. The economic burden of Fe deficiency anemia is particularly significant in low- and middle-income countries, where nutritional deficiencies are compounded by socio-economic factors, inadequate healthcare access, and limited food resources [9]. Addressing this deficiency requires comprehensive strategies, including dietary diversification, supplementation, and public health initiatives aimed at accessibility of Fe-rich foods. As countries strive to meet global health targets, the role of Fe supplementation and fortification programs in reducing the prevalence of Fe deficiency and its associated health risks must be prioritized [8,9].

Research has shown that foliar application with Fe-based nanoparticles at concentrations between 4 and 400 mg/L could boost plant growth, photosynthetic efficiency, and stress tolerance. For example, in wheat (*Triticum aestivum* L.), 400 mg/L iron oxide nanoparticles led to an increased grain yield of 3776 kg/ha [10]. Likewise, dragonhead (*Dracocephalum moldavica* L.) showed larger leaf size and greater biomass upon treatment with 60 mg/L iron oxide nanoparticles [11]. Soybean (*Glycine max* L.) demonstrated a 40.1% increase in seed yield under drought conditions and a 32.6% increase under well-watered conditions when treated with 200 mg/L of magnetite nanoparticles (nFe_3_O_4_) [12]. Broad bean (*Vicia faba* L.) exhibited a 35% growth in plant height through foliar application of 100 mg/L nFe_3_O_4_ [13], whereas green bean (*Phaseolus vulgaris* L.) demonstrated a 37% boost in total biomass after receiving treatment with 50 mg/L of hematite nanoparticles (nFe_2_O_3_) [14]. Moreover, our recent study found that applying 500–1000 mg Fe/L as nFe_3_O_4_ to kidney bean (*Phaseolus vulgaris* L.) during drought resulted in yield increases of 29–72%, enhancements in grain quality—shown by increased protein content of 7–17% and Fe content of 10–45%—and significant improvements in photosynthetic efficiency (245–259% for chlorophyll-a and 203–260% for chlorophyll-b) [15].

A previous study demonstrated improved photosynthesis in green beans with foliar application of 200 mg/L iron nanoparticles, iron sulfate, and iron chelate, leading to an 18%, 17%, and 6% increase in total chlorophyll, respectively [14]. The impact on antioxidants and various biochemical processes also relied on the dosage used. For instance, broad beans with 100 mg/L nFe_3_O_4_ treatment led to a 37% increase in indole-3-acetic acid, 29% increase in gibberellic acid, and 25% increase in proteins, while abscisic acid dropped by 44% [13]. In green beans, the activity of nitrate reductase increased by about 71–72% when suitable concentrations (50 mg/L) of nFe_2_O_3_ were used [14]. The use of 50 mg/L nFe_2_O_3_ led to the highest levels of flavonoid content and non-enzymatic antioxidant activity in *Dracocephalum kotschyi* (Boiss.) [16]. Moderate doses—generally ranging from 25 to 200 mg/L of nFe_2_O_3_ or similar compounds—yielded optimal results, while excessively high concentrations were toxic to plants [14,17].

Shallot (*Allium hirtifolium* Boiss.) is a biennial herbaceous plant belonging to the Amaryllidaceae family, predominantly found in the semi-arid mountainous regions of Iran, the Mediterranean, and parts of Central Asia, at altitudes ranging from 2700 to 3000 m with annual precipitation of 300–500 mm and temperatures between 11 and 14 °C [18,19]. Recognized for its distinctive flavor profile, its bulbs (sister bulbs) and leaves are used in various culinary applications, including salads and pickles, as well as in traditional medicine for treating rheumatic and inflammatory ailments [20]. The plant is rich in bioactive compounds like allicin and organosulfides, which contribute to its notable antimicrobial and antioxidant activities. It also provides abundant fatty acids and vital minerals, including potassium, sodium, magnesium, iron, copper, zinc, and manganese [21]. These properties make Persian shallot an important candidate for dietary inclusion and potential therapeutic applications, especially in the management of diabetes and infections, lowering blood lipid levels, preventing arteriosclerosis, and aiding in blood pressure regulation [18,22].

Shallot offers considerable nutritional value because of its strong antioxidant qualities. Earlier research has shown that micronutrient nanoparticles, in addition to their natural nutritional benefits, can stimulate the synthesis of secondary metabolites in plants [16,23]. Furthermore, employing nanoscale technologies has demonstrated improved plant resilience under unfavorable environmental conditions, serving as a novel method for growing crops that can tolerate climate-induced stress with improved yield [24]. Considering the challenges plants encounter in obtaining Fe from semi-arid soils with high pH levels [25,26] and that areas suitable for shallot farming frequently encounter cold weather, in this field study conducted in two separate semi-arid regions with disparate soil types, we sought to improve shallot production and overall plant performance via bulb-priming followed by foliar application of magnetite nanoparticles (nFe_3_O_4_). Thus, we hypothesized that the use of nFe_3_O_4_, as a source of Fe micronutrients, would promote cellular and morpho-physiological performance, bulb yield, antioxidant activities, and iron fortification in shallot grown in semi-arid regions.

## 2. Results

### 2.1. Magnetite Nanoparticle Characterization

**Figure 1:**
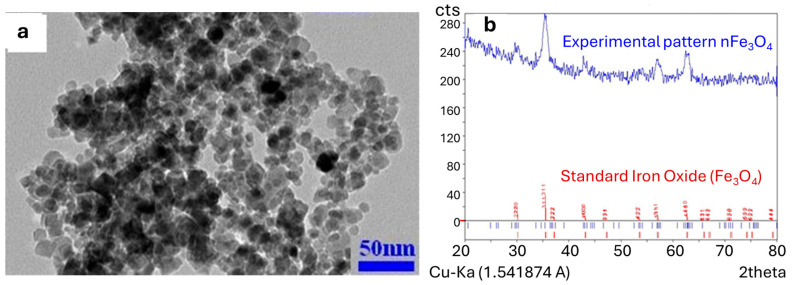
Electron micrograph (transmission electron microscopy) (**a**) and X-ray diffraction pattern of magnetite nanoparticles (nFe_3_O_4_) used in this study (**b**).

The purity and crystalline structure of the nanoparticles were assessed via X-ray diffraction (XRD, EQUINOX, INEL; INEL Inc., Stratham, NH, USA) analysis over a 2θ range of 20–80°, confirming the consistent crystal structure of nFe_3_O_4_. The average particle size was determined using transmission electron microscopy (TEM; Philips EM 208; Thermo Fisher Scientific, Waltham, MN, USA). Dynamic light scattering (DLS) was employed to evaluate the hydrodynamic diameter (HDD) and zeta potential of the nanoparticles in aqueous suspension. TEM imaging revealed that the spherical nFe_3_O_4_ nanoparticles had a uniform diameter in the range 20–30 nm (Figure 1). Additionally, DLS showed an average HDD of 368 ± 16 nm and zeta potential of −17.2 ± 0.43 mV for nFe_3_O_4_ in aqueous suspension.

### 2.2. Effects on Leaf Subcellular Structures

**Figure 2:**
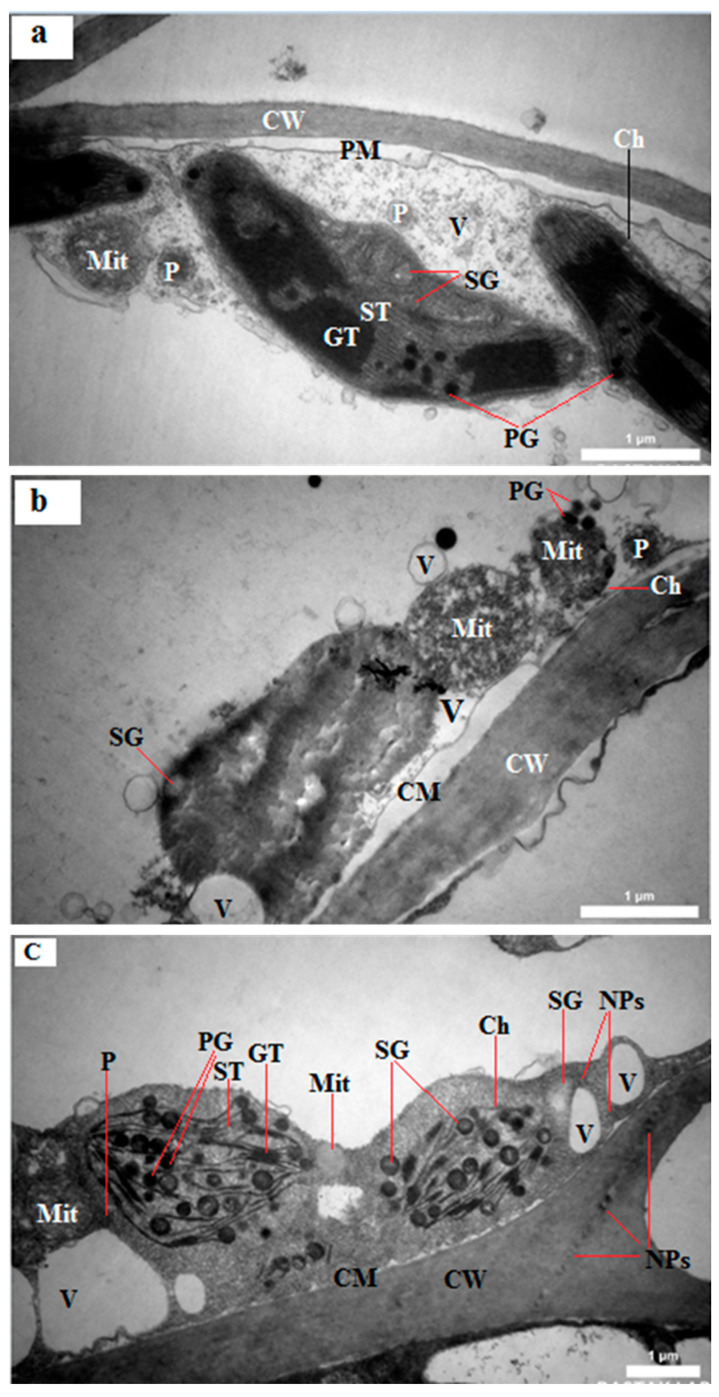
Ultrastructure of shallot leaf cells. (**a**) Control; (**b**) 1800 mg Fe/L FeSO_4_; (**c**) 900 mg Fe/L magnetite nanoparticles (nFe_3_O_4_). Ch: chloroplast; Mit: mitochondria; SG: starch grain; CW: cell wall; CM: cell membrane; PM: plasma membrane; ST: stroma thylakoid; GT: granum thylakoid; P: peroxisome, V: vacuole, PG: plastoglobuli; NPs: nanoparticles.

In untreated control plant leaves, cell walls were relatively thin, while chloroplasts appeared elongated with compact thylakoid grana, and a limited number of plastoglobuli and distinct mitochondria were observed (Figure 2a). For plants treated with FeSO_4_ (1800 mg/L), cell walls appeared thin to medium thickness, chloroplasts were smaller, thylakoids were somewhat indistinct, mitochondria appeared enlarged, plastoglobuli mostly gathered around the mitochondria, and relatively few starch grains were observed (Figure 2b). In plants treated with nFe_3_O_4_ (900 mg/L), cell walls appeared thick, chloroplasts were clearly defined with well-structured thylakoid grana and stroma, mitochondria appeared normal, starch grains were numerous, plastoglobuli were found around the chloroplasts, and nanoparticles were detected within the cell wall and cytoplasm (Figure 2c). It is, however, unclear whether the nanoparticles were composed of Fe as elemental analysis could not be performed.

### 2.3. Effects on Chlorophyll

**Figure 3:**
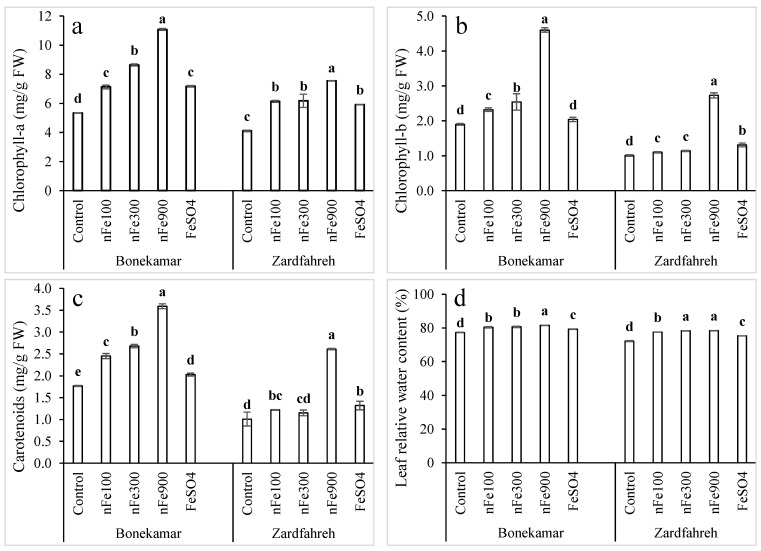
Effects of magnetite nanoparticles (nFe_3_O_4_) on chlorophyll-a (**a**), chlorophyll-b (**b**), carotenoid (**c**), and leaf relative water content (**d**) in shallots grown in Bonekamar and Zardfahreh sites. Means with different letters at each location indicate statistically significant differences based on LSD test (*p* ≤ 0.05). Concentrations are in mg Fe/L for respective compounds. Error bar denotes standard deviation (±SD).

ANOVA revealed that Fe fertilization significantly influenced chlorophyll-a content (Table S1). At both study sites, chlorophyll-a levels increased markedly with rising concentrations of nFe_3_O_4_. At the Bonekamar site, chlorophyll-a content reached 11.1 mg/g FW with 900 mg/L nFe_3_O_4_ treatment, which was 108% greater than the control chlorophyll-a content (5.3 mg/g FW; Figure 3a). At Zardfahreh, the chlorophyll-a level was 7.6 mg/g FW compared to 4.11 mg/g FW in the control (Figure 3a). At the Bonekamar site, chlorophyll-a levels under 100 mg/L nFe_3_O_4_ were comparable to those observed with 1800 mg/L FeSO_4_ (*p* > 0.05); however, in Zardfahreh, there was no significant difference among the 100 mg/L and 300 mg/L nFe_3_O_4_ treatments and the 1800 mg/L FeSO_4_ treatment (*p* > 0.05; Figure 3a).

A significant effect of Fe fertilizer on chlorophyll-b content was observed across both locations according to ANOVA results (Table S1). In Bonekamar, 900 mg/L nFe_3_O_4_ treatment led to the highest chlorophyll-b concentration (4.6 mg/g), representing increases of 142% and 126% relative to the control and 1800 mg/L FeSO_4_ treatments, respectively (*p* < 0.05; Figure 3b). However, no significant difference in chlorophyll-b content was observed between the control and 1800 mg/L FeSO_4_ treatments at this location (*p* > 0.05). At the Zardfahreh site, 900 mg/L nFe_3_O_4_ treatment yielded the highest chlorophyll-b content (2.73 mg/g), which was 171% and 108% greater than the control and 1800 mg/L FeSO_4_ treatments, respectively (*p* < 0.05; Figure 3b).

### 2.4. Effects on Carotenoids

The carotenoid content was significantly influenced by the application of Fe fertilizers (Table S1). At the Bonekamar site, carotenoid levels with 100 and 300 mg/L nFe_3_O_4_ treatments were 21% and 32% higher, respectively, than those observed with 1800 mg/L FeSO_4_ treatment (*p* < 0.05); however, no significant difference was observed between these treatments at the Zardfahreh site (*p* > 0.05; Figure 3c). Specifically, carotenoid concentrations at 900 mg/L nFe_3_O_4_ were 3.59 mg/g in Bonekamar and 2.61 mg/g in Zardfahreh, representing increases of 77% and 97%, respectively, relative to the 1800 mg/L FeSO_4_ treatments at both sites (Figure 3c).

### 2.5. Effects on Leaf Relative Water Content

The pattern of relative water content (RWC) changes across fertilizer treatments was largely consistent between the two locations (Figure 3d). Nonetheless, the RWC range at Bonekamar was 77–82%, whereas at Zardfahreh, RWC ranged from 72 to −78%. Although treatment with 1800 mg/L FeSO_4_ promoted RWC by 2.6–4.3% compared to control, plants treated with nFe_3_O_4_ had a significantly higher RWC increase (1.3–4.1%) compared to the 1800 mg/L FeSO_4_ treatment (*p* < 0.05; Figure 3d). 

### 2.6. Effects on Electrolyte Leakage

**Figure 4:**
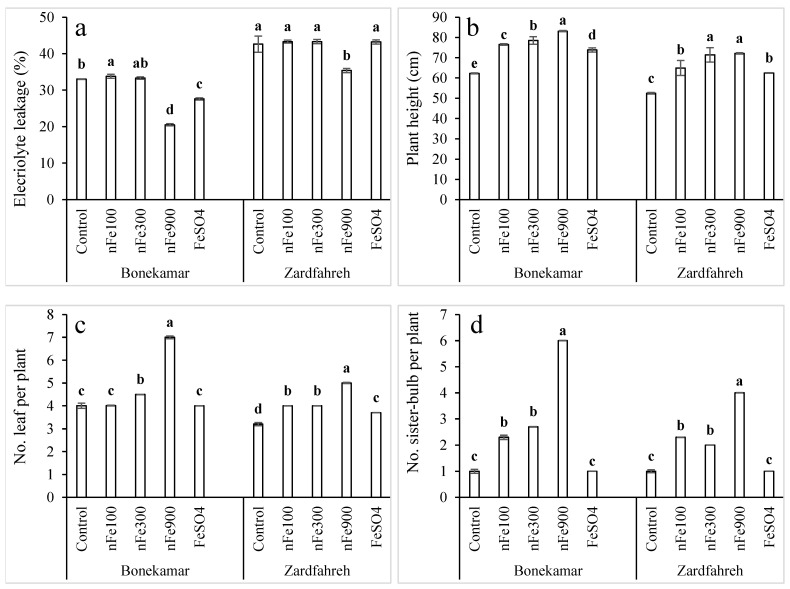
Effects of magnetite nanoparticles (nFe_3_O_4_) on electrolyte leakage (**a**), plant height (**b**), number of leaves per plant (**c**), and number of sister bulbs per plant (**d**) of shallots grown in Bonekamar and Zardfahreh sites. Means with different letters at each location indicate statistically significant differences based on LSD test (*p* ≤ 0.05). Concentrations are in mg Fe/L for respective compounds. Error bar denotes standard deviation (±SD).

At the Zardfahreh site, apart from the 900 mg/L nFe_3_O_4_ treatment, no significant difference was detected between the iron treatments and the control (*p* > 0.05); however, the 900 mg/L nFe_3_O_4_ treatment led to a 17% reduction in electrolyte leakage when compared to the control (*p* < 0.05; Figure 4a). At the Bonekamar site, 100 mg/L nFe_3_O_4_ resulted in a 2.3% increase in electrolyte levels relative to the control, whereas the 900 mg/L nFe_3_O_4_ and 1800 mg/L FeSO_4_ treatments significantly decreased these levels compared to the control, with reductions of 38% and 16%, respectively. Additionally, at this site, the 900 mg/L nFe_3_O_4_ treatment also caused a 20% decrease in electrolyte leakage when compared to the 1800 mg/L FeSO_4_ (*p* < 0.05; Figure 4a).

### 2.7. Effects on Plant Height

Iron treatments had significant effects on plant height at both study sites (Table S2). The greatest plant height was observed at the 900 mg/L nFe_3_O_4_ concentration, with increases ranging from 33% to 38% compared to control (*p* < 0.05; Figure 4b). At the Bonekamar site, a plant height under 1800 mg/L FeSO_4_ was 3.5–11.2% lower than that observed with nFe_3_O_4_ treatments. Conversely, at the Zardfahreh site, 1800 mg/L FeSO_4_ resulted in a significantly lower plant height compared to nFe_3_O_4_ at 300–900 mg/L (*p* < 0.05; Figure 4b).

### 2.8. Effects on Leaf Number

Results showed that Fe fertilizers had significant effects on the number of leaves per plant at both locations (Table S2). At the Bonekamar site, the 900 mg/L nFe_3_O_4_ treatment yielded the highest leaf count, averaging seven leaves per plant, which corresponded to a 75% increase over the control (*p* < 0.05). At the Zardfahreh site, this treatment resulted in a 59% increase in leaf count relative to the control (Figure 4c). At the Bonekamar site, leaf number increased significantly with 300–900 mg/L nFe_3_O_4_ treatments compared to 1800 mg/L FeSO_4_, while at Zardfahreh, all nFe_3_O_4_ treatments produced significantly greater numbers of leaves compared to 1800 mg/L FeSO_4_ (*p* < 0.05; Figure 4c).

### 2.9. Effects on Sister Bulb Number

The number of sister bulbs per plant was significantly affected by Fe fertilizer application in both locations (Table S2). While 1800 mg/L FeSO_4_ did not elicit a significant response, nFe_3_O_4_ concentrations of 100 and 300 mg/L increased the sister bulb count per plant by 100–167% relative to the control (*p* < 0.05; Figure 4d). At 900 mg/L nFe_3_O_4_, the number of sister bulbs per plant was 500% and 300% greater than the control at the Bonekamar and Zardfahreh sites, respectively (*p* < 0.05; Figure 4d).

### 2.10. Effects on Bulb Diameter

**Figure 5:**
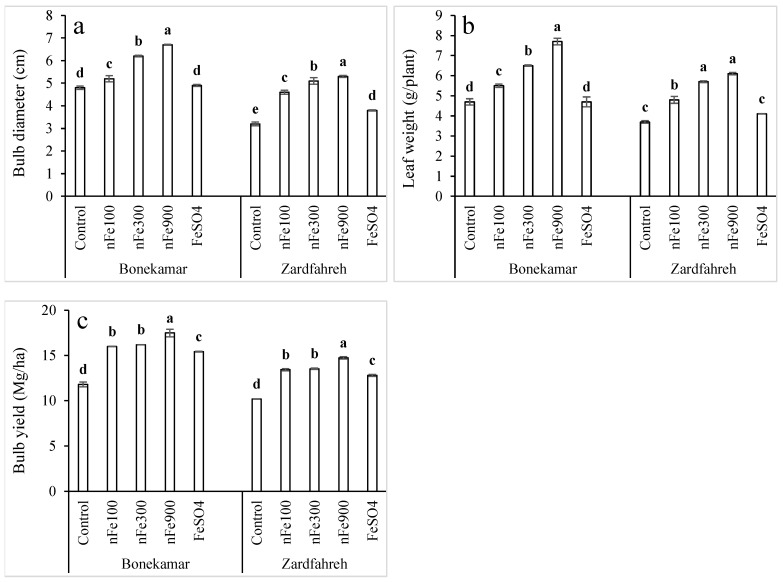
Effects of magnetite nanoparticles (nFe_3_O_4_) on bulb diameter (**a**), leaf weight (**b**), and bulb yield (**c**) in shallots grown in Bonekamar and Zardfahreh sites. Means with different letters at each location indicate statistically significant differences based on LSD test (*p* ≤ 0.05). Concentrations are in mg Fe/L for respective compounds. The error bar denotes standard deviation (±SD).

At the Bonekamar site, the bulb diameter with 1800 mg/L FeSO_4_ treatment did not differ significantly from the control (Figure 5a). Conversely, at the Zardfahreh site, the bulb diameter with 1800 mg/L FeSO_4_ treatment was significantly greater, exhibiting a 17.3% increase compared to control (*p* < 0.05). Application of nFe_3_O_4_ at all tested concentrations (100, 300, and 900 mg/L) resulted in significant increases in bulb diameter compared to 1800 mg/L FeSO_4_ treatment. Specifically, at Bonekamar, these increases were 5.9%, 27.3%, and 38.2%, respectively, while at Zardfahreh site, the corresponding increases were 20.1%, 34.4%, and 38.8%, respectively, compared to FeSO_4_ treatment (*p* < 0.05; Figure 5a).

### 2.11. Effects on Leaf Weight

No significant differences were observed in leaf fresh weight in plants treated with 1800 mg/L FeSO_4_ and the control. However, nFe_3_O_4_ treatments led to a significant increase in leaf fresh weight compared to control and 1800 mg/L FeSO_4_ (*p* < 0.05; Figure 5b). At the Bonekamar site, leaf fresh weight increased by 15–17%, 36–38%, and 63–65% at 100, 300, and 900 mg/L nFe_3_O_4_ concentrations, respectively, compared to 1800 mg/L FeSO_4_ treatment or control. Similarly, at the Zardfahreh site, increases of 18–29%, 39–52%, and 48–62% were observed at the corresponding concentrations, compared to 1800 mg/L FeSO_4_ treatment or control (*p* < 0.05; Figure 5b).

### 2.12. Effects on Bulb Yield

Fe fertilizations had significant effects on bulb yield across both study locations (Table S2). The highest bulb yield was recorded with the 900 mg/L nFe_3_O_4_ treatment, reaching 17.5 Mt/ha at Bonekamar and 14.7 Mt/ha at the Zardfahreh site. These yields represent increases of 13.4% and 15.1% over the 1800 mg/L FeSO_4_ treatment, and 48.2% and 44.7% relative to the control, respectively (*p* < 0.05; Figure 5c). Furthermore, 1800 mg/L FeSO_4_ treatment also significantly improved bulb yield compared to control, with increases ranging from 25.8% to 30.7% for the Zardfahreh and Bonekamar sites, respectively. Bulb yields at lower (100 and 300 mg/L) nFe_3_O_4_ treatments also exceeded 1800 mg/L FeSO_4_ treatment; bulb yield was 551–766 kg/ha at the Bonekamar site and 621–728 kg/ha at the Zardfahreh site (*p* < 0.05; Figure 5c).

### 2.13. Effects on Bulb Total Phenol Content

**Figure 6:**
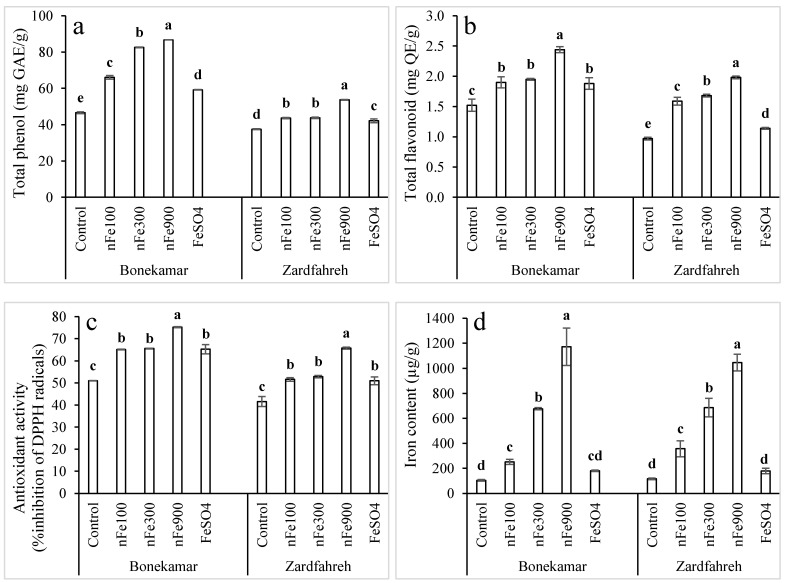
Effects of magnetite nanoparticles (nFe_3_O_4_) on total phenol content (**a**), total flavonoid content (**b**), antioxidant activity (**c**), and iron content (**d**) in shallot bulbs grown in Bonekamar and Zardfahreh sites. Means with different letters at each location indicate statistically significant differences based on LSD test (*p* ≤ 0.05). Concentrations are in mg Fe/L for respective compounds. The error bar denotes standard deviation (±SD).

At both sites, total bulb phenol content exhibited a dose-dependent increase in response to increasing concentrations of nFe_3_O_4_. At the Bonekamar site, total phenol levels in nFe_3_O_4_-treated samples (66–87 mg gallic acid/g) were 12–46% and 42–86% higher than those observed with the 1800 mg/L FeSO_4_ treatment and control, respectively (*p* < 0.05; Figure 6a). At the Zardfahreh site, total phenol content ranged from 37.5 to 53.7 mg gallic acid/g, with the highest concentration recorded at the 900 mg/L nFe_3_O_4_ treatment. This value was 27.4% and 43.2% greater compared to the 1800 mg/L FeSO_4_ treatment and control, respectively (*p* < 0.05; Figure 6a).

### 2.14. Effects on Bulb Total Flavonoid Content

At the Bonekamar and Zardfahreh sites, total bulb flavonoids increased in a dose-dependent manner, while the highest total flavonoid content was recorded at 2.44 mg quercetin/g and 1.98 mg quercetin/g, respectively, upon treatment with 900 mg/L nFe_3_O_4_ (Figure 6b). At Bonekamar, no significant difference was observed among 100 mg/L and 300 mg/L nFe_3_O_4_ treatments and 1800 mg/L FeSO_4_ treatment. At the Zardfahreh site, total bulb flavonoids with 1800 mg/L FeSO_4_ treatment were 17.5% higher than control, whereas with nFe_3_O_4_ treatments, total bulb flavonoids increased by 39–73% and 64–104% compared to 1800 mg/L FeSO_4_ and control, respectively (*p* < 0.05; Figure 6b).

### 2.15. Effects on Bulb Antioxidant Activity

The shallot bulb antioxidant activity (measured as free radical inhibition %) was influenced by the application of Fe fertilizers (Table S3). Maximum antioxidant activity at both the Bonekamar and Zardfahreh sites was attained with 900 mg/L nFe_3_O_4_ treatment, demonstrating a significant enhancement relative to both the control and the 1800 mg/L FeSO_4_ treatments (*p* < 0.05; Figure 6c). No significant difference in antioxidant activity was observed among the 100 mg/L and 300 mg/L nFe_3_O_4_ treatments and the 1800 mg/L FeSO_4_ treatment across both locations (*p* > 0.05; Figure 6c). 

### 2.16. Effects on Bulb Fe Fortification

A significant dose-dependent enrichment in bulb Fe fortification (uptake) was observed with nFe_3_O_4_ treatments at both the Bonekamar and Zardfahreh sites (Figure 6d). At 900 mg/L nFe_3_O_4_ treatment in the Bonekamar and Zardfahreh sites, bulb Fe levels were the highest, ranging from 8206 to 7332 μg Fe/g DW (Figure 6d). At the Zardfahreh site, 900 mg/L nFe_3_O_4_ treatment led to a 486% increase in bulb Fe levels compared to 1800 mg/L FeSO_4_ treatment (*p* < 0.05). At Bonekamar, 300 mg/L and 900 mg/L nFe_3_O_4_ treatments increased bulb Fe by 275% and 549%, respectively, compared to 1800 mg/L FeSO_4_ treatment. However, at both sites, bulb Fe content with 1800 mg/L FeSO_4_ treatment did not differ significantly from control (*p* < 0.05; Figure 6d).

A comparison of the evaluated parameters at the two sites showed that shallots grown in Bonekamar exhibited lower electrolyte leakage than those in Zardfahreh (Table S4). However, the number of sister bulbs per plant and the iron content in the bulbs were similar between the two locations (Table S4). Additionally, the average values of other morphological, physiological, and biochemical traits, along with bulb quality, were significantly higher in shallots cultivated in Bonekamar compared to those in Zardfahreh (Table S4).

The Pearson correlation matrix (Table 1) reveals changes in morpho-physiological and yield-related variables. In both sites, bulb yield and bulb quality variables frequently showed strong correlation coefficients. Additionally, bulb yield was positively correlated with morphological and physiological parameters (excluding electrolyte leakage), as well as bulb quality. Furthermore, antioxidant capacity exhibited a strong positive correlation with all parameters except for electrolyte leakage (Table 1).

Economic analysis revealed that even though nanofertilizers are significantly more expensive than ferrous sulfate (Table S5), the increased bub yield they generate offsets the higher fertilizer cost. As a result, the profit gained from increased bulb yield with nFe_2_O_3_ treatments was approximately USD 480 to USD 1260 greater than that from using ferrous sulfate at both sites (Table S5).

## 3. Discussion

As a crucial micronutrient, Fe is known to promote plant growth, yield, and the production of secondary metabolites in various crops [27,28,29]. Fe is vital for numerous physiological processes in plants, including DNA synthesis, respiration, photosynthesis, and protein synthesis. Conversely, in semi-arid soils that are mainly calcareous, Fe deficiency is closely linked to several soil characteristics, including elevated pH, high salinity, reduced organic matter, and the presence of free calcium carbonate [15,25]. Emerging nano-micronutrient-based fertilizer studies have shown promise in circumventing this limitation, while showing potential for improved growth and yield in agricultural crops, particularly in semi-arid areas [15,30,31].

The evidence of prominent chloroplasts featuring clear thylakoids (Figure 2c) suggests the potential for improved photosynthetic activities by nFe_3_O_4_ (Figure 3a,b), and its carboxylation leads to starch production, consequently promoting plant growth (Figure 4 and Figure 5). Relatively large vacuoles suggest a proper moisture level in the leaf, while normal mitochondria can also signify healthy foliar respiration by leaves in plants treated with nFe_3_O_4_. Conversely, leaves of plants subjected to ferrous sulfate may exhibit decreased photosynthesis and increased respiration because of smaller chloroplasts and larger mitochondria (Figure 2c).

Chlorophylls are crucial pigments involved in photosynthesis that play a key role in converting light energy to chemical energy [32]. Leaf chlorophylls and carotenoids serve as primary indicators defining plant physiological and photosynthetic performance [33,34]. Carotenoids are active pigments that function as antioxidants, safeguarding chlorophyll from oxidation and photodegradation [33,35]. In the current study, improvements in leaf chlorophyll and carotenoid content were documented in shallots exposed to nFe_3_O_4_ compared to those exposed to FeSO_4_. A putative explanation is that nFe_3_O_4_ may have promoted iron oxygen reductase activity, which could increase porphyrin metabolism to generate 5-aminolevulinic acid, a chlorophyll precursor [36]. In this context, Tombuloglu et al. [37] indicated that 500 mg/L nFe_3_O_4_ greatly enhanced the carotenoid content in tomato leaves (*Solanum lycopersicum*). A comparable result was reported by Shahzad et al. [38], indicating that the foliar application of nFe_3_O_4_ at 100 mg/L elevated chlorophyll levels in bell pepper (*Capsicum annuum*) leaves compared to control. This could be attributed to the positive correlation between photosynthesis rate and chlorophyll content [15], thereby enhancing the photosynthetic efficiency of shallot plants through nFe_3_O_4_ applications. A previous study indicated that plants utilize Fe^2+^/Fe^3+^ released from the nFe to improve the functionality of the photosynthetic machinery [39]. In our study, the use of 900 mg/L nFe_3_O_4_ could remarkably improve shallot photosynthetic pigment levels, overall vegetative growth, and Fe fortification.

In shallots treated with nFe_3_O_4_, leaf RWC exceeded that of plants treated with FeSO_4_, which could be linked to improved root performance and water absorption under nFe_3_O_4_ treatments. Our results showed that the subterranean bulbs and above-ground parts of shallot functioned not only to preserve but also improve the RWC in leaves. Shallot leaves with optimal RWC can enhance crop production. Studies have shown that foliar application of nFe_3_O_4_ (50 to 1000 mg/L) in kidney bean led to increased RWC under both drought and non-drought conditions, relative to the respective controls [15]. Likewise, application of 200 mg/L nFe_3_O_4_ in soybean, under both drought and non-drought scenarios, led to increases of 15.8 and 10% in RWC, respectively, versus untreated plants [12].

The current research indicated that the highest values of vegetative growth measurements, such as plant height, leaf count, leaf weight, bulb size, and number of sister bulbs, were recorded in shallot treated with nFe_3_O_4_, followed by FeSO_4_, compared to untreated plants (Figure 4a–d and Figure 5a). The pronounced positive effects for the above parameters were documented in shallots that were bulb-primed and foliarly treated with 900 mg/L nFe_3_O_4_. Moreover, the current research indicated that the application of nFe_3_O_4_ resulted in the development of a consistent intracellular structure featuring comparatively thick cell walls, large vacuoles, sizable chloroplasts, normal mitochondria, thylakoid formations, and more starch granules (Figure 2c). Consequently, the beneficial outcomes of employing nFe_3_O_4_ at concentrations of 100–900 mg/L resulted in a notable increase in bulb size and yield at both sites (Figure 5a,c).

The comparable pattern of changes in bulb Fe augmentation in shallot bulbs alongside plant growth parameters further validates the direct influence of nFe_3_O_4_ on plant performance and micronutrient Fe fortification. Consequently, the enhancement in vegetative growth metrics may result from the rise in chlorophyll levels, photosynthesis rates, and absorption of additional nutrients from the soil, all of which substantially boost the accumulation of polysaccharides and organic material in various plant parts. These results are consistent with the findings reported by Mahmoud et al. [13], which demonstrated that the foliar application of 100 mg/L of nFe_3_O_4_, iron chelate, and iron sulfate fertilizer increased plant height (35, 26, and 20%, respectively); leaf area (39, 18, and 8%, respectively); and dry weight (53, 37, and 11%, respectively) in beans, compared to control. Studies using perennial ryegrass (*Lolium perenne* L.), pumpkin (*Cucurbita mixta*), and maize (*Zea mays* L.) have also demonstrated enhanced root length with nFe treatments [29,40]. Konate et al. [41] demonstrated that nFe can engage with plants and produce free OH radicals, which may promote the breakdown of pectin in the plant cell wall, thereby softening the root cell wall to facilitate plant root development as well as water absorption via osmosis. Dong et al. [42] demonstrated that nFe_3_O_4_ can influence root growth patterns via processes like carbon reallocation or modifications in the cell wall. The emergence of sister bulbs alongside the main bulb indicates stimulatory effects of nFe_3_O_4_ treatments, which promote bulb size and yield (Figure 5a,c). Shallot bulbs develop in the ground, where diseases originating in the soil are commonly found. According to findings from prior research, employing nanoparticles could enhance bulb growth by inhibiting soil-borne diseases [43]. This is particularly crucial for farmers, who experience a significant delay in harvesting shallots or do not collect certain bulbs for resowing the following year. Thus, bulb-priming and foliar application of nFe_3_O_4_ could circumvent this age-old agricultural issue and prevent significant economic losses, particularly for small-scale farmers.

At the Bonekamar site, applying 100–900 mg/L of nFe_3_O_4_ led to significantly higher phenolic and flavonoid levels in shallot bulbs compared to FeSO_4_ (Figure 6a,b). Nonetheless, plants treated with FeSO_4_ also showed increased phenolic and flavonoid levels compared to control plants. Applying nFe_3_O_4_ to the bulbs and leaves of shallot plants could enhance nutrient absorption and utilization, source-sink capacity, and photosynthetic efficiency, leading to the rise in secondary metabolites in this study. In *Hypericum perforatum*, 50 mg/L nFe_2_O_3_ improved secondary metabolite production, particularly furohyperforin (1.3 times) and miquelianin (5.2 times) [34]. Likewise, the use of 50 mg/L nFe_2_O_3_ led to the greatest flavonoid content and non-enzymatic antioxidant activity in *Dracocephalum kotschyi* [16]. In *Solidago virgaurea*, the essential oil and flavonoid (quercetin and rutin) levels in plants treated with five applications of low-dose nFe_3_O_4_ (1 mg/L) showed a significant increase [44]. Foliar nFe_3_O_4_ applications (20–60 mg/L) also improved total phenolic compounds in wheat [45].

The rise in antioxidant capacity of shallot bulb extracts subjected to nFe_3_O_4_ treatment suggests a positive association between nFe availability and antioxidant activity. Certain secondary compounds with significant antioxidant capabilities were enhanced by 900 mg/L of nFe_3_O_4_, leading to the peak antioxidative potential in shallot bulbs grown in both locations. Previous studies have also indicated the benefits of nanoparticles in boosting antioxidant potential and the functioning of both enzymatic and non-enzymatic antioxidants in nanoparticle-treated plants [15,35,46]. For instance, the foliar application of nFe_3_O_4_ not only elevated antioxidant enzyme activity but also vitamin C, glutathione, and antioxidant capacity in radish (*Raphanus sativus*) leaves and roots by 24–147% [28]. Additionally, Zuluaga et al. [26] indicated that Fe fertilizer successfully enhanced Fe fortification in edible plant tissues, boosting their nutritional value.

The fortification of Fe in shallot bulbs correlates with increasing nFe_3_O_4_ concentrations and is notably greater than that of FeSO_4_, suggesting that nFe_3_O_4_ could be uptaken through the leaf and bulb surfaces (Figure 6d). The presence of Fe in bulbs enhances stomatal opening, leading to greater CO_2_ absorption and enzyme activity in chloroplasts, which subsequently enhances photosynthetic efficiency [38,47]. Numerous investigations have demonstrated comparable reactions of FeNPs across various crops [48]. Concentrations of nFe_2_O_3_ at 50–200 mg/L and 200–500 mg/L markedly enhance the Fe levels in *Dracocephalum kotschyi* and wheat leaves, respectively [16,46]. The higher accumulation of Fe in the bulbs of shallot treated with nFe fertilizers, as opposed to FeSO_4_, could be linked to unique characteristics of nFe, known for their reduced surface area, enhanced absorption, and superior binding capabilities compared to ionic Fe forms [41,49]. Elevated Fe levels in shallot bulbs could positively affect the reduction in abscisic acid (ABA), a crucial plant hormone that inhibits growth and metabolism, and promote senescence. Researchers have observed the lowest ABA levels in bean leaf treated with 100 mg/L of nFeO [13]. Moreover, the oral intake of shallot bulbs enriched with Fe can significantly supply dietary Fe for individuals suffering from severe Fe deficiency.

Although the bulb yield and other growth parameters follow similar trends due to nFe_3_O_4_ treatments in both locations, the yield in Zardfahreh is around 16% less than in Bonekamar (Table S4). Zardfahreh’s sandy loam soil is not as fertile as Bonekamar’s clay loam soil when considering organic matter, nutrients, and moisture retention (Table 2 and Table 3). Table 2 shows that the organic matter levels in Zardfahreh soil are less than half of Bonekamar soil, while its salinity is twice as high (measured as EC). As a result, the lack of organic matter and water holding ability, along with relatively higher soil salinity, can significantly reduce the yield potential of shallots in Zardfahreh (Table S4). Lasmini et al. [50] showed that in sandy loam soil, the addition of nutrients and organic matter raised soil organic matter from 0.89 to 2.43%, which doubled the yield of shallots. Additionally, Rahayu et al. [51] noted that incorporating organic matter into the soil could alleviate the negative impacts of salinity while promoting plant growth, increasing bulb yield, and improving bulb size. Furthermore, it was observed that after sowing shallot bulbs, the minimum temperatures in the Zardfahreh region were lower (1–1.3 °C), indicating that the photosynthesis rate might have been lower. Nonetheless, the highest temperatures in both areas displayed no considerable differences (Table 2). Zardfahreh’s lower elevation, along with increased relative humidity—especially during the bulb growth and filling stage (April to June)—could have resulted in lower photosynthetically active solar radiation accessible to plants, affecting overall shallot growth parameters.

Research indicates varying degrees of toxicity associated with iron-based nanoparticles. For instance, Zhu et al. [52] found that excessive accumulation of iron oxide nanoparticles could lead to oxidative stress and DNA damage in zebrafish. However, another study noted no to low cytotoxicity to human lung cells upon exposure to iron oxide nanoparticles, indicating that they may not pose significant health risks at realistic exposure levels [53]. Likewise, Coccini et al. [54] documented the low toxicity of magnetite nanoparticles in human mesenchymal stem cells compared to other nanomaterials composed of toxic heavy metals. Further, the fate of iron nanoparticles in shallots subjected to cooking and/or during digestion remains unknown, making it unreasonable to predict potential acute and chronic toxicity risks in humans. Therefore, while there maybe potential for risk, particularly with high-dose ingestion, the current scientific evidence does not permit a definitive conclusion, thus necessitating future focused studies investigating the fate of iron-based nanoparticles in shallots upon being subjected to cooking and during digestion.

## 4. Materials and Methods

### 4.1. Material Preparation

Magnetite nanoparticles (nFe_3_O_4_) and ferrous sulfate (ionic Fe, FeSO_4_) were procured from the Nanosany and Kargozar companies in Iran, respectively. Shallot (*A. hirtifolium* Fereydounshahr landrace) bulbs were obtained as a courtesy of local farmers. Bulbs weighing approximately 20–25 g were selected and treated with a fungicide, Carbendazim, for surface disinfection. Subsequently, the bulbs were stored at 15 °C until planting.

### 4.2. Experimental Design and Treatment

This study includes annual field experiments conducted separately in two semi-arid sites in Iran: Bonehkamar site (49°58′ N, 32°55′ E, 2524 m asl.; Isfahan Province) and Zardfahreh site (49°48′ N, 33°0′ E, 2391 m asl.; Isfahan Province). Two separate sites/regions were chosen because of their contrasting soil types and properties: Bonehkamar had clay loam, higher organic carbon (OC), and lower salinity/electrical conductance (EC) versus Zardfahreh that presented sandy loam, lower OC, and higher EC. Table 2 summarizes the meteorological data for the duration of the experiments.

Each experiment followed a randomized complete block design with three replications. The experimental treatments consisted of various concentrations of nFe_3_O_4_ (100, 300, and 900 mg Fe/L), a concentration of 1800 mg Fe/L ferrous sulfate (FeSO_4_), and an untreated control group (distilled water). The selection of 1800 mg Fe/L FeSO_4_ was based on recommended soil Fe fertilizer levels, while the lower concentrations of nFe_3_O_4_ were chosen at 6%, 17%, and 50% of the FeSO_4_ concentration to investigate if at lower Fe concentrations nFe_3_O_4_ would demonstrate comparable or superior performance compared to the recommended ionic Fe level. These treatments were applied through bulb-priming before sowing, followed by foliar spraying during the vegetative stage at 194 days post-planting. Nanoparticle solutions were prepared to the desired concentrations, subjected to sonication and homogenization, and evenly sprayed on the bulbs. For the purpose of bulb-priming, the bulbs were uniformly sprayed until thoroughly moistened and then stored unwashed in plastic bags until planting. Bulb-priming was found to enhance root development at the time of planting. Furthermore, foliar application was performed using hand sprayers calibrated to deliver a volume corresponding to 400 L per hectare.

Soil samples were collected from each location in early October for soil testing to determine fertilizer requirements. Table 3 displays the characteristics of the soil at the two semi-arid sites.

**Figure 7:**
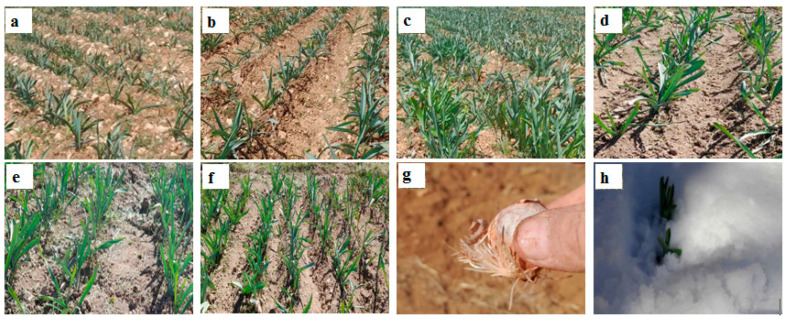
Representative photographs showing the effect of iron fertilizers on shallots growth in the Bonekamar (**a**–**c**) and Zardfahreh (**d**–**f**) sites. (**a**,**d**) Control; (**b**,**e**) 1800 mg Fe/L FeSO_4_; (**c**,**f**) 900 mg Fe/L magnetite nanoparticles (nFe_3_O_4_); (**g**) enhancing root development prior to planting through bulb-priming; (**h**) shallots sprouting under the winter snow.

The land was deeply plowed with a moldboard plow and harrowed with a disc harrow, followed by the application of 30 Mt/ha of rotted sheep manure and chemical fertilizer based on the soil test recommendations. Experimental plots were established after ridge preparation. Bulbs were planted on October 22 and 30 in Zardfahreh and Bonekamar, respectively, at a depth of 15–20 cm with row spacing of 50 cm and plant spacing of 10 cm. Each plot consisted of 5 planting lines, each 2 m in length. Weed control and soil cultivation were conducted in early spring. Plants were irrigated with natural spring water twice after spring rain ceased. Figure 7 displays the phenotype of shallots treated with 900 mg/L nFe_3_O_4_ and 1800 mg/L FeSO_4_ at the Bonekamar and Zardfahreh sites.

Pre-harvest measurements included plant height, leaf count, leaf weight, relative leaf water content, and photosynthetic pigment levels. At harvest, bulb diameter, sister bulb count, phenolic and flavonoid compound levels, antioxidant activity, and iron fortification in the bulbs were assessed through random sampling.

### 4.3. Measurement of Traits

#### 4.3.1. Subcellular Structure

The investigations of leaf organelles and possible alterations in (sub)cellular structure were conducted using a transmission electron microscope (TEM) (Philips EM208S, 100 KV; Amsterdam, The Netherlands). At 220 days after planting, leaf samples were randomly gathered from plants grown in control, 1800 mg Fe/L FeSO_4_, and 900 mg Fe/L magnetite nanoparticles treatments, and the leaves were immersed in a 1:1 solution of final Spurr resin and absolute acetone at room temperature for one hour. Subsequently, the samples were transferred to a 1:3 mixture of the final resin and absolute acetone for three hours, followed by an overnight immersion in the final resin solution. The samples were then heated at 70 °C in Eppendorf tubes containing Spurr resin for over 9 h. Afterward, the specimens were sectioned using a microtome (Leica EM UC7; Leica Microsystems, Boston, MA, USA), and the sections were stained with alkaline lead citrate and uranyl acetate for 10 min before being examined under the TEM.

#### 4.3.2. Vegetative Traits and Yield

At 220 days post-planting, underground and aerial parts of six plants were randomly harvested from each plot, and after transferring the samples to the laboratory, the ensuing traits were recorded: plant height was measured using a meter scale, the number of leaves and sister bulbs were counted, and bulb diameter was measured with a caliper. Leaves were separated and placed at 70 °C until the moisture content was stabilized, then they were weighed. To determine the bulb yield at the Bonekamar and Zardfahreh sites, at 225 and 220 days post-planting, respectively, an area of 2 m^2^ of each plot was harvested, and the bulbs (main bulb and sister bulb) were weighed and reported as tons per hectare.

#### 4.3.3. Photosynthetic Pigments

The quantification of photosynthetic pigments was conducted following the methodology described by Lichtenthaler and Buschmann [55]. At 220 days post-planting, 250 mg of fresh leaf tissue was thoroughly ground in a porcelain mortar with 10 mL of 80% acetone to obtain a homogeneous solution. The mixture was centrifuged at 3500 rpm for 10 min, after which the supernatant was collected and volume adjusted to 10 mL with 80% acetone. Absorbance measurements were recorded at wavelengths of 663.2 nm, 646.8 nm, and 470 nm using an UV-Vis spectrophotometer (AE-UV 1606; A & E Lab (UK) Co., Ltd., London, England); 80% acetone served as a blank. The photosynthetic pigment concentrations were calculated using established equations and expressed as mg/g of fresh tissue weight, where A represents the absorbance of the extract at the specified wavelengths.

(1) $$Chlorophyll\;a\left(\frac{mg}{mL}\right)=12.5\left(A663.2\right)-2.79\left(A646.8\right)$$

(2) $$Chlorophyll\;b\left(\frac{mg}{mL}\right)=21.51\left(A646.8\right)-5.1\left(A663.2\right)$$

(3) $$Carotenoids\;(mg/mL)=\frac{1000(A470)-1.82(Chl.a)-85.02(Chl.b)}{198}$$

#### 4.3.4. Leaf Relative Water Content

Leaf samples were randomly collected at 220 days post-planting, and the relative water content (RWC) was determined according to Martinez et al. [56]. The RWC was calculated using Equation (4):

(4) $$\mathrm{RWC}\;(\%)\;=\;(\frac{FW-DW}{SW-BW})\;\times \;100$$

where FW denotes the fresh weight of the leaf immediately after sampling, DW represents the dry weight after oven drying, and SW corresponds to the saturated weight following rehydration in distilled water.

#### 4.3.5. Electrolyte Leakage

The stability of the cell membrane was assessed using the membrane electrolyte leakage (EL) method, as outlined by Dionisio-Sese and Tobita [57]. The EL was calculated using the following equation:

(5) $$\mathrm{EL}\;(\%)\;=\;\frac{C1}{C2}\;\times \;100$$

C1 = initial electrical conductivity. C2 = final electrical conductivity.

#### 4.3.6. Antioxidant Activity

Methanolic extracts were prepared via a soaking extraction method. Briefly, 100 mL of 70% methanol was added to 10 g of shallot bulb and maintained in the dark for 48 h. The mixture was filtered to separate solid pulp, and the filtrate was allowed to evaporate at room temperature to remove methanol, as described by Gasmi et al. [58]. For the antioxidant assay, 1 mL of the methanol extract was mixed with 1 mL of 0.1 mM DPPH solution (prepared by dissolving 4 mg of DPPH radical in 100 mL methanol). The control consisted of 1 mL of pure methanol in place of the extract, and pure methanol also served as a blank. After incubation in the dark for 30 min, absorbance was measured at 517 nm using a spectrophotometer (AE-UV 1606; A & E Lab (UK) Co., Ltd., London, England). Free radical inhibition % was calculated following Equation (6) [59]:

(6) $$\mathrm{Free}\;\mathrm{radical}\;\mathrm{inhibition}\;(\%)\;=\;(\frac{AC-AS}{AC})\;\times \;100$$

where AC and AS represent the absorbance values of the control and the sample, respectively. The resulting values correspond to the % inhibition of DPPH radicals by the methanol extract.

#### 4.3.7. Total Phenol

Total phenol content was determined employing the Folin–Ciocalteu method [60]. Specifically, 0.01 g of shallot bulb extract was dissolved in 60% methanol and diluted to a final volume of 10 mL. Subsequently, 0.1 mL of this solution was transferred to a test tube, to which 0.5 mL of 10% Folin–Ciocalteu reagent was added. After an incubation period of 5 min, 0.4 mL of 7.5% sodium carbonate solution was added. The mixture was then incubated in dark for 30 min, and absorbance was recorded at 765 nm. A calibration curve was generated using gallic acid standards ranging from 0.01 to 0.1 mg/mL.

#### 4.3.8. Total Flavonoid

Adhering to the protocol by Marinova et al. [60], the total flavonoid content was estimated in the shallot bulbs. Briefly, 5 mL of methanol was added to 500 mg of fresh bulb samples. The extract was agitated in a shaker for 24 h, then centrifuged at 6000 rpm for 10 minutes. The supernatant was collected and transferred into new tubes for further analysis. Then 1 mL of the prepared extract was combined with 4 mL of distilled water, after which 300 µL of 5% sodium nitrite was added. Five minutes post sodium nitrite addition, 600 µL of 10% aluminum chloride was introduced, and after six minutes, 4 mL of 0.5 N sodium hydroxide was added. Sample absorbance was measured at 510 nm. A standard calibration curve was constructed using various concentrations of quercetin, and sample flavonoids are expressed as mg quercetin FW/g.

#### 4.3.9. Determination of Bulb Iron Content

At 220 days post-planting, bulb samples were collected to quantify iron fortification in shallot bulbs using Inductively Coupled Plasma Optical Emission Spectrometry (ICP-OES; SPECTRO Analytical Instruments GmbH, Kleve, Germany). Briefly, 10 mL of concentrated nitric acid (70%) was added to the dried bulb tissue samples, followed by incubation at 150 °C for 1 h. Upon cessation of boiling, 2 mL of perchloric acid was introduced, and the mixture was maintained at 215 °C for 2 h. Subsequently, the samples were cooled under a fume hood. Next, 10 mL of deionized water heated to 90 °C was added, and the mixture was thoroughly homogenized and cooled. The resulting suspension was filtered through Whatman No. 42 filter paper and adjusted to a final volume of 25 mL. Total Fe concentration in the filtrate was quantified via ICP-OES and expressed as µg Fe/g dry bulb weight [15,61].

### 4.4. Statistical Analysis

Experimental data were analyzed using SAS software version 9.1. Initially, Bartlett’s test was conducted to assess the homogeneity of variances between the two locations. Due to detected heterogeneity of variances, separate analysis of variance (ANOVA) was performed for each location, and mean comparisons were conducted using the Least Significant Difference (LSD) test at a 5% significance level.

## 5. Conclusions

Taken together, we demonstrated that bulb-priming followed by foliar application of nFe_3_O_4_ fertilizer could significantly promote overall morpho-physiological performance, bulb size and yield, secondary metabolites, antioxidant activities, and Fe fortification in shallot in (both) semi-arid regions. All tested concentrations of nFe_3_O_4_ had a beneficial effect on overall plant growth and performance, with 900 mg/L showing the greatest effects. Improved photosynthetic apparatus, secondary metabolites, and antioxidative activities coupled with observed thickened cell walls facilitating water retention could have played a role in the improved shallot performance with nFe_3_O_4_ treatments, particularly at 900 mg/L. The achieved increase of up to 500% Fe fortification in bulbs (8.20 mg Fe/g DW) via the application of 900 mg/L nFe_3_O_4_ is a breakthrough for human intervention to address global Fe deficiency that severely affects 30% of the world population. These results indicate that bulb-priming followed by foliar application of nFe_3_O_4_ may serve as a novel approach for improving shallot production in semi-arid regions.

## Figures and Tables

**Table 1:** Pearson correlation matrix for the analyzed variables of shallot plants as a function of iron fertilizers at two semi-arid sites.

	Cha	Chb	Car	RWC	EL	PH	LN	SN	BD	LW	BY	TP	TF	AA	IC
Cha		0.92 **	0.97 **	0.88 **	−0.75 **	0.90 **	0.90 **	0.92 **	0.93 **	0.95 **	0.87 **	0.94 **	0.95 **	0.92 **	0.96 **
Chb	0.76 **		0.9 **	0.73 **	−0.83 **	0.73 **	0.99 **	0.97 **	0.87 **	0.92 **	0.68 **	0.77 **	0.89 **	0.79 **	0.92 **
Car	0.77 **	0.99 **		0.88 **	−0.67 **	0.87 **	0.92 **	0.97 **	0.94 **	0.98 **	0.83 **	0.92 **	0.93 ***	0.87 **	0.96 **
RWC	0.89 **	0.47 ^ns^	0.51 ^ns^		−0.47 ^ns^	0.97 **	0.65 **	0.78 **	0.79 **	0.82 **	0.96 **	0.92 **	0.88 **	0.94 **	0.76 **
EL	−0.61 *	−0.94 **	−0.91 **	−0.35 ^ns^		−0.54 *	−0.86 **	−0.72 **	−0.56 *	−0.62 *	−0.53 *	−0.47 ^ns^	−0.78 **	−0.70 **	−0.70 **
PH	0.87 **	0.52 *	0.52 *	0.89 **	−0.41 ^ns^		0.66 **	0.76 **	0.78 **	0.81 **	0.99 **	0.92 **	0.89 **	0.96 **	0.79 **
LN	0.95 **	0.88 **	0.89 **	0.83 **	−0.79 **	0.80 **		0.95 **	0.85 **	0.90 **	0.61 *	0.73 **	0.87 **	0.79 **	0.93 **
SN	0.82 **	0.83 **	0.84 **	0.72 **	−0.80 **	0.68 **	0.94 **		0.90 **	0.94 **	0.71 **	0.82 **	0.90 **	0.79 **	0.94 **
BD	0.87 **	0.57 *	0.59 *	0.96 **	−0.48 ^ns^	0.90 **	0.87 **	0.82 **		0.98 **	0.72 **	0.94 **	0.82 **	0.74 **	0.97 **
LW	0.64 *	0. 50 ^ns^	0.47 ^ns^	0.68 **	−0.54 *	0.62 *	0.68 **	0.63 *	0.69 **		0.76 **	0.93 **	0.87 **	0.79 **	0.97 **
BY	0.97 **	0.65 *	0.68 **	0.95 **	−0.51 ^ns^	0.88 **	0.91 **	0.78 **	0.91 **	0.67 *		0.89 **	0.88 **	0.96 **	0.73 **
TP	0.93 **	0.92 **	0.93 **	0.77 **	−0.82 **	0.77 **	0.99 **	0.92 **	0.82 **	0.65 *	0.88 **		0.85 **	0.86 **	0.90 **
TF	0.87 **	0.89 **	0.70 **	0.93 **	−0.60 *	0.85 **	0.93 **	0.92 **	0.97 **	0.71 **	0.90 **	0.89 **		0.93 **	0.88 **
AA	0.95 **	0.88 **	0.88 **	0.80 **	−0.78 **	0.80 **	0.98 **	0.89 **	0.84 **	0.67 **	0.91 **	0.98 **	0.88 **		0.79 **
IC	0.82 **	0.80 **	0.80 **	0.79 **	−0.76 **	0.78 **	0.92 **	0.90 **	0.90 **	0.72 **	0.80 **	0.91 **	0.93 **	0.89 **	

Cha: chlorophyll-a; Chb: chlorophyll-b; Car: carotenoids; RWC: relative water content; EL: electrolyte leakage; PH: plant height, LN: number of leaves per plant; SN: number of sister bulbs per plant; BD: bulb diameter; LW: leaf weight; BY: bulb yield; TP: total phenol; TF: total flavonoid; AA: antioxidant activity; IC: iron content; Gray shading: Bonekamar site; Unshaded: Zardfahreh site; ns, non-significant; * significant at *p* ˂ 0.05; and ** significant at *p* ˂ 0.01; and *** significant at *p* ˂ 0.001.

**Table 2:** Monthly air temperature, daylight length, relative humidity, and precipitation during the study period (October 2023 through October 2024 at both study locations).

	Air Temperature (°C)						Relative Humidity (%)		Rainfall (mm)	
Month	Min.		Max.		Mean					
	B	Z	B	Z	B	Z	B	Z	B	Z
October	8.8	9.2	21.2	21.6	15.0	15.4	31.5	33.5	3.8	5.3
November	3.8	2.8	15.3	15.1	9.4	9.0	46.5	51.0	52.9	68.8
December	−0.4	−1.1	11.5	11.8	5.6	5.4	35.0	39.0	16.1	18.5
January	−0.9	−2.2	8.4	8.7	3.8	3.3	43.5	50.0	6.5	10.5
February	−3.6	−4.7	5.7	7.0	1.0	1.2	51.5	56.5	90.0	129
March	−3.5	−4.0	6.3	7.9	1.4	2.0	52.5	56.0	133	75.7
April	1.5	1.1	12.9	13.0	7.2	7.05	46.0	52.5	90.4	154
May	6.2	5.4	16.7	17.2	11.4	11.3	49.5	53.5	96.1	86.2
June	10.9	10.1	25.6	25.8	18.2	17.95	34.0	38.0	7.1	10.6
July	15.4	14.3	30.9	30.4	23.2	22.35	25.0	26.5	0.6	0.0
August	17.7	16.4	32.9	32.8	25.3	24.6	22.5	24.5	1.7	0.0
September	12.9	13.1	28.3	29.4	20.6	21.25	22.5	24.0	0.5	0.0
October	8.9	8.5	22.3	22.7	15.6	15.6	29.0	31.5	3.0	0.0
November	4.3	3.6	13.8	13.9	9.0	8.75	47.0	51.0	32.7	62.9

B: Bonekamar; Z: Zardfahreh.

**Table 3:** Soil properties at study locations.

Parameter	Unit	Bonekamar	Zardfahreh
Texture		Clay loam	Sandy loam
EC	µS/cm	600	1200
pH	-	7.8	8.2
Total nitrogen	g/kg	1.3	0.5
Phosphorus #	mg/kg	12	6.0
Potassium #	mg/kg	220	180
OC	g/kg	12	5.0
Iron #	mg/kg	3.5	2.8
Zinc #	mg/kg	0.25	0.18
Copper #	mg/kg	0.8	0.5
Manganese #	mg/kg	4.5	3.2

EC: electrical conductance; OC: organic carbon; #: available form.

## Data Availability

The original contributions presented in this study are included in the article. Further inquiries can be directed to the corresponding authors.

## Supplementary Materials

The following supporting information can be downloaded at https://www.mdpi.com/article/10.3390/plants15020279/s1, Table S1: Mean square (MS) from the analysis of variance (ANOVA) performed for photosynthetic pigments, leaf relative water content (RWC), and electrolyte leakage in shallot influenced by iron fertilizers at two semi-arid sites; Table S2: Mean square (MS) from the analysis of variance (ANOVA) performed for plant height, number of leaves per plant, number of sister-bulb per plant, bulb diameter, leaf weight, and bulb yield in shallot influenced by iron fertilizers at two semi-arid sites; Table S3: Mean square (MS) from the analysis of variance (ANOVA) performed for total phenol, total flavonoid, antioxidant activity, and iron content in shallot influenced by iron fertilizers at two semi-arid sites; Table S4: Mean, standard deviation, *t*- and *p*-values for studied traits of shallot at two semi-arid sites; Table S5: Economic analysis of iron nanoparticle fertilization of shallot grown at Bonekamar and Zardfahreh sites.
