# Supplementary material for: Bulb-Priming Followed by Foliar Magnetite Nanoparticle Applications Improve Growth, Bulb Yield, Antioxidant Activities, and Iron Fortification in Shallot in Semi-Arid Regions
Source: Plants (Basel). 2026 Jan 16;15(2):279. doi: 10.3390/plants15020279 (PMC12845011; doi:10.3390/plants15020279)
Supplement: Supplementary file 1 [file plants-15-00279-s001.zip › plants-4081102-supplementary.pdf]

Supporting Information

Bulb-Priming Followed by Foliar Magnetite Nanoparticle Applications Improve Growth, Bulb Yield, Antioxidant Activities, and Iron Fortification in Shallot in Semi-arid Regions

Soroush Moguee [1], Sina Fallah [1,*], Lok R. Pokhrel [2,*] and Zohrab Adavi [3]

[1] Department of Agronomy, Faculty of Agriculture, Shahrekord University, Shahrekord 115, Iran
[2] Department of Public Health, The Brody School of Medicine, East Carolina University, Greenville, NC, USA
[3] Department of Agriculture, Payame Noor University, Tehran, Iran
* Correspondence: pokhrell18@ecu.edu (L.R.P.); falah1357@yahoo.com (S.F.)

Table S1. Mean square (MS) from the analysis of variance (ANOVA) performed for photosynthetic pigments, leaf relative water content (RWC), and electrolyte leakage in shallot influenced by iron fertilizers at two semi-arid sites.

S.O.V.[a]	DF[b]	Chlorophyll-a	Chlorophyll-b	Carotenoids	RWC	Electrolyte leakage
**Bonekamar site**						
Replication	2	0.006^{ns}	0.02^{ns}	0.001^{ns}	0.06^{ns}	0.11^{ns}
Iron fertilizer	4	13.8**	3.64**	1.49**	7.64**	97.7**
Error	8	0.005	0.01	0.002	0.07	0.13
CV (%)		0.89	4.06	1.87	0.33	1.22
**Zardfahreh site**						
Replication	2	0.04^{ns}	0.001^{ns}	0.02^{ns}	0.02^{ns}	1.93^{ns}
Iron fertilizer	4	4.52**	1.56**	1.28**	20.8**	36.4**
Error	8	0.04	0.002	0.01	0.02	1.04
CV (%)		3.52	3.23	5.36	0.17	2.46

[a] S.O.V.: Source of Variation; [b] Degrees of Freedom; ns, non-significant; ** significant at $p < 0.01$.

Table S2. Mean square (MS) from the analysis of variance (ANOVA) performed for plant height, number of leaves per plant, number of sister-bulb per plant, bulb diameter, leaf weight, and bulb yield in shallot influenced by iron fertilizers at two semi-arid sites.

S.O.V.[a]	DF[b]	Plant height	Leaf number	Sister-bulb number	Bulb diameter	Leaf weight	Bulb yield
**Bonekamar site**							
Replication	2	1.68^{ns}	0.005^{ns}	0.001^{ns}	0.01^{ns}	0.08*	0.005
Iron fertilizer	4	182**	4.99**	12.57**	2.22**	5.01**	13.7
Error	8	0.82	0.003	0.17	0.004	0.01	0.06
CV (%)		1.21	1.17	15.7	1.22	1.71	1.61
**Zardfahreh site**							
Replication	2	16.4^{ns}	0.001^{ns}	0.07^{ns}	0.02^{ns}	3.95**	0.02^{ns}
Iron fertilizer	4	192**	1.36**	4.57**	2.24**	2.98**	8.58**
Error	8	11.8	0.001	0.07	0.005	0.12	0.01
CV (%)		5.31	0.64	12.5	1.57	7.21	0.65

[a] S.O.V.: Source of Variation; [b] Degrees of Freedom; ns, non-significant; * significant at $p < 0.05$; and ** significant at $p < 0.01$.

Table S3. Mean square (MS) from the analysis of variance (ANOVA) performed for total phenol, total flavonoid, antioxidant activity, and iron content in shallot influenced by iron fertilizers at two semi-arid sites.

S.O.V.[a]	DF[b]	Total phenol	Total flavonoid	Antioxidant activity	Iron content
**Bonekamar site**					
Replication	2	0.32[ns]	0.004[ns]	0.45[ns]	263494[ns]
Iron fertilizer	4	827**	0.32**	225**	29444166**
Error	8	0.25	0.006	0.96	210885
CV (%)		0.74	4.13	1.52	13.8
**Zardfahreh site**					
Replication	2	0.03[ns]	0.002[ns]	2.93[ns]	39472[ns]
Iron fertilizer	4	105**	0.51**	222**	22142959**
Error	8	0.38	0.001	1.63	167752
CV (%)		1.40	2.02	2.43	12.2

[a] S.O.V.: Source of Variation; [b] Degrees of Freedom; ns, non-significant; ** significant at $p < 0.01$.

Table S4. Mean, standard deviation, t- and p-values for studied traits of shallot at two semi-arid sites.

Parameters	Bonekamar site (n=15)		Zardfahreh site (n=15)		t-value	P-value
	Mean	Std. Deviation	Mean	Std. Deviation		
Chlorophyll-a	7.87	1.98	5.98	1.15	3.19	0.0035*
Chlorophyll-b	2.68	1.02	1.46	0.67	3.86	0.0006*
Carotenoids	2.51	0.65	1.46	0.61	4.53	0.0001*
Relative water content	79.8	1.49	76.3	2.44	4.73	<.0001*
Electrolyte leakage	32.6	5.29	43.4	3.36	7.36	<.0001*
Pant height	74.9	7.27	64.7	7.99	3.65	0..0011*
Leaf number	4.72	1.20	3.96	0.62	2.19	0.0397*
Sister-bulb number	2.60	1.92	2.07	1.16	0.92	0.3653
Bulb diameter	5.57	0.80	4.39	0.80	4.03	0.0004*
Leaf weight	5.83	1.20	4.88	1.22	2.15	0.0407*
Bulb yield	15.4	1.99	12.9	1.57	3.74	0.0008*
Total phenol	68.2	15.4	44.2	5.49	5.71	<.0001*
Total flavonoid	1.94	0.31	1.47	0.38	3.70	0.0009*
Antioxidant activity	64.4	8.05	52.6	8.05	4.03	0.0004
Iron content	3339	2928	3341	2535	0.00	0.9982

(*) Indicates a significant difference between the two sites.

Table S5. Economic analysis of iron nanoparticle fertilization of shallot grown at Bonekamar and Zardfahreh sites.

	**Bonekamar**				**Zardfahreh**			
**Treatment**	**nFe100**	**nFe300**	**nFe900**	**$FeSO_4$**	**nFe100**	**nFe300**	**nFe900**	**$FeSO_4$**
Bulb yield over control (kg/ha)	4174	4389	5681	3623	3243	3350	4554	2622
Cost of fertilizer ($)	91.1	273	821	22.2	91.1	273	821	22.2
Cost of foliar spray ($)	7.1	7.1	7.1	7.1	7.1	7.1	7.1	7.1
Profit ($)	4076	4110	4853	3594	3145	3070	3726	2593
